# From Normal Flora to Brain Abscesses: A Review of *Streptococcus intermedius*

**DOI:** 10.3389/fmicb.2020.00826

**Published:** 2020-05-07

**Authors:** Elio Issa, Tamara Salloum, Sima Tokajian

**Affiliations:** Department of Natural Sciences, School of Arts and Sciences, Lebanese American University, Byblos, Lebanon

**Keywords:** *Streptococcus intermedius*, brain abscess, virulence, *Streptococcus anginosus* group (SAG), fibronectin (FN), laminin

## Abstract

*Streptococcus intermedius* is a β-hemolytic Gram-positive member of the *Streptococcus anginosus* group (SAG). Despite being a part of the normal microbiota, it is one of the most common pathogens associated with brain and liver abscesses and thoracic empyema, increasing as a result the morbidity and mortality rates in affected patients. Though there are numerous published case reports on *S. intermedius* infections, it is still understudied compared to other SAG members. Our knowledge of the genomic factors contributing to its dissemination to the brain and abscess development is also limited to few characterized genes. In this review, we summarize our current knowledge on *S. intermedius* identification methods, virulence factors, and insight provided by the whole-genome and correlate patients’ metadata, symptoms, and disease outcome with *S. intermedius* infections in 101 recent case reports obtained from PubMed. This combined information highlights the gaps in our understanding of *S. intermedius* pathogenesis, suggesting future research directions to unveil the factors contributing to abscess development.

## Introduction

*Streptococcus intermedius* is a β-hemolytic Gram-positive coccus and member of the *Streptococcus anginosus* group (SAG), also referred to as the “*Streptococcus milleri*” (Facklam, 2002). Members of the SAG are part of the normal microbiota and are found at various mucosal sites in the respiratory, gastrointestinal, and genitourinary tracts with variable carriage levels (Gossling, 1988). However, members of the SAG are medically important because they are frequently encountered in invasive suppurative infections in numerous body sites, causing liver and brain abscesses, dentoalveolar infections, and infective endocarditis (Jacobs et al., 1995). Members of the SAG have been identified as the most common organism being isolated from brain (Prasad et al., 2006; Sibley et al., 2012) and liver abscesses (Sibley et al., 2010) and thoracic empyema (Ahmed et al., 2006). Moreover, they elicit pulmonary exacerbation and worsen the disease outcome in patients with cystic fibrosis (Parkins et al., 2008). *S. anginosus* has been associated with intra-abdominal and gastrointestinal tract infections, whereas *S. intermedius* has been isolated more frequently from purulent head and neck specimens and central nervous system infections (Whiley et al., 1992; Bantar et al., 1996; Clarridge et al., 2001). An association between *S. constellatus* and thoracic infections has been previously suggested (Jacobs et al., 1995). Two more recent studies that investigated possible associations between species of SAG bacteria and specific clinical syndromes on 245 and 76 bacterial isolates, respectively, did not show any significant correlation between the species and the site of infection (Siegman-Igra et al., 2012; Junckerstorff et al., 2014). The only association the authors found was a higher relative representation of *S. constellatus* among blood culture isolates (Siegman-Igra et al., 2012). Very few studies also evaluated SAG bacterial infections and clinical outcomes, including morbidity and mortality, with varying conclusions (Jacobs et al., 1994, 1995; Casariego et al., 1996; Siegman-Igra et al., 2012; Junckerstorff et al., 2014). Mortality rates among patients with SAG-associated bacteremia range between 10 and 16%. Jacobs et al. (1994) examined 19 cases of SAG-associated bacteremia and found that 78.9% (*n* = 15) were caused by *S. anginosus* of which 33.3% (*n* = 5) died. In another study, they observed a 16% mortality rate among patients with SAG-associated bacteremia (Jacobs et al., 1995). Casariego et al. (1996) reviewed 30 cases of SAG-associated bacteremia and observed a 10% (*n* = 3) mortality rate among patients infected with *S. constellatus*, while 6.7% (*n* = 2) of patients were infected with *S. anginosus* and survived. On the other hand, Siegman-Igra et al. (2012) found that *S. anginosus* accounted for 67.8% (*n* = 19) of 28 SAG-associated bacteremia with a 15.8% (*n* = 3) mortality. It is noteworthy that patients with invasive *S. intermedius* infections had significantly longer hospital stays compared to patients infected with *S. anginosus* and significantly higher mortality rates than patients infected with *S. constellatus* (Junckerstorff et al., 2014).

*S. intermedius* bacteremia and liver abscesses have been frequently reported in patients following recent dental manipulation. Since *S. intermedius* is part of the commensal oral flora in humans, dental cleaning can result in bacteremia and seeding of the liver via the hematogenous route even in the absence of an active oral infection (Livingston and Perez-Colon, 2014).

The taxonomic grouping of SAG members has long been debatable (Coykendall et al., 1987), and this is partly due to varying nomenclature (Facklam, 2002) and partly due to the low resolution provided by traditional phenotypic identification methods (Jones, 1978; Kilian et al., 1989; Whiley et al., 1990, 1999). More recently, it has been shown that the SAG consists of three distinct species: *S. anginosus*, *S. intermedius*, and *S. constellatus* (Jensen et al., 2013). *S. constellatus* was further divided into three subspecies (subsp *constellatus*, subsp *pharynges*, and subsp *viborgensis*), and *S. anginosus* was divided into two subspecies (subsp *anginosus* and subsp *whileyi*) based on the use of seven core housekeeping genes (Jensen et al., 2013).

Despite being phenotypically diverse, members of the SAG share few common characteristics, such as having a slow growth rate, a distinctive “caramel smell,” the ability to hydrolyze arginine, the ability to produce acetoin from glucose, and the inability to ferment sorbitol (Gossling, 1988). They also have a variable Lancefield serogrouping; a system of grouping streptococci based on antigenic differences in C carbohydrates located in their cell wall. *S. anginous* are typically Lancefield types A, C, F, or G, *S. constellatus* are typically Lancefield types C, F, or no antigen, and *S. intermedius* are generally not typeable using the Lancefield method. Almost half of all human SAG clinical isolates are of the Lancefield F type (Grinwis et al., 2010).

**FIGURE 1:**
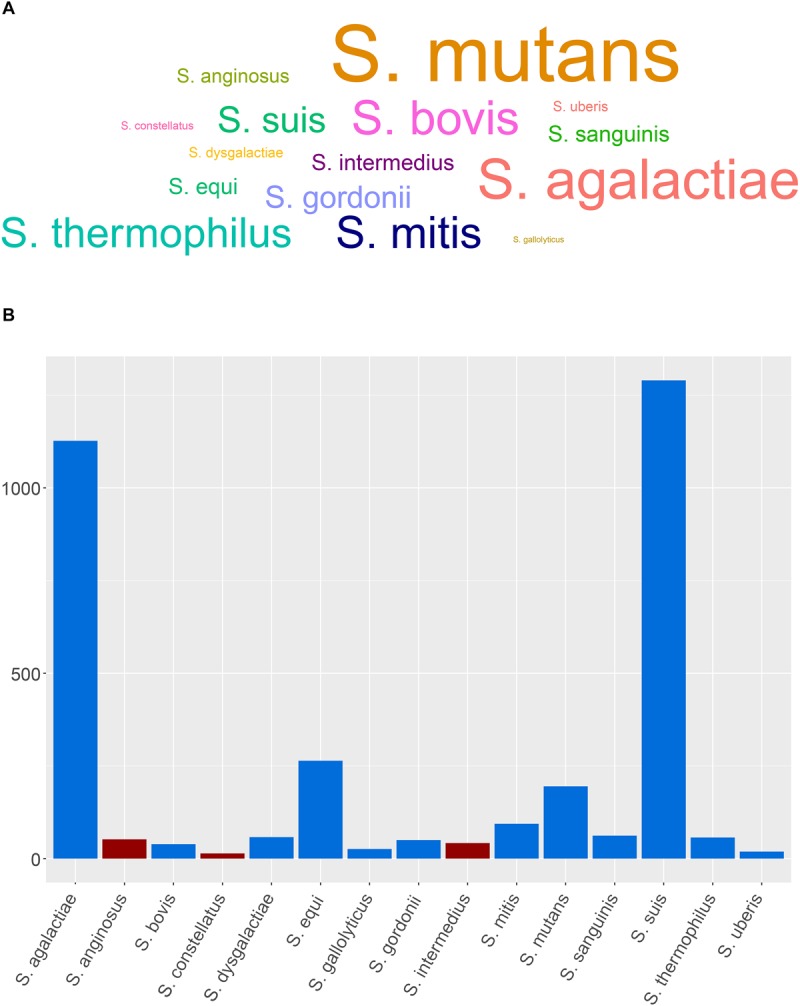
Representative members of streptococci and SAG members and the distribution of the genome assemblies. **(A)** Word cloud showing the medically relevant streptococci and SAG members based on a PubMed Central data (last accessed: July 3rd, 2019). **(B)** Distribution of the genome assemblies on NCBI for SAG members and other medically relevant streptococci (last accessed on 08/05/2019); SAG members are depicted in red.

A search on PubMed Central revealed that SAG members are clearly underrepresented compared to other medically relevant streptococci with the least represented being on *S. constellatus* followed by *S. anginosus* and *S. intermedius* (Figure 1A). Very few recent studies were published correlating clinical outcomes, morbidity, and mortality in SAG-infected patients, while the prediction of the disease outcome in *S. intermedius*-infected patients remains unrealistic. Also, the advent of whole-genome sequencing (WGS) has provided in-depth information into *S. intermedius* genome organization, virulence, and secretion systems that might help in highlighting specific genetic markers and single point variants in various SAG members, as demonstrated in the work done by Issa et al. (2019). Understanding the various genetic components contributing to abscess development could also help in finding novel targets for therapy and vaccine development. Given the caveats in our understanding of *S. intermedius* pathobiology, prediction of disease outcome, and genome content, this review has summarized our current knowledge on the *S. intermedius* pathogen, its various identification methods, known virulence factors, and regulation of gene expression involved in abscess development and whole-genome provided insights. Additionally, we have summarized 101 recent case reports of *S. intermedius* infections with full description of patients’ metadata, clinical profiles, symptoms, neurological results, imaging results, isolation sites, co-infections, abscess localization, prescribed antibiotics to treat the infection, and disease outcome obtained from PubMed in an attempt to find patterns associated with the infection. This combined information highlights the gaps in our understanding of *S. intermedius* pathogenesis and suggests future research directions to unveil the factors contributing to abscess development.

## Identification

Due to the lack of reliable phenotypic differences between members of the SAG, these streptococci are rarely identified to the species level in clinical microbiological laboratories. One of the basic phenotypic characteristics for clinical laboratories is their reaction on blood agar plates. Hemolysis is used as a guide for managing patients and gives hints into the classification of the bacterium to the species level (Facklam, 2002). One step further in the identification and speciation of the SAG members could be performed using commercially available identification kits, such as API 20 Strep (bioMérieux, Lyon, France).

Real-time PCR, mass spectrometry (MALDI-TOF-MS), as well as conventional PCR assays were also used to differentiate between individual species of the SAG (Takao et al., 2004; Friedrichs et al., 2007; Desar et al., 2008; Olson et al., 2010; Reißmann et al., 2010). The PCR amplification covering an 819 bp fragment of the *ily* gene encoding intermedilysin and its 3’-flanking region was shown to be specific for *S. intermedius* in contrast to other SAG members (Goto et al., 2002). Other target genes included 16S rRNA, *cpn60*, and *rpoB* (Olson et al., 2013). These, however, might not provide enough discriminatory power between closely related species. For instance, 16S rRNA gene-based phylogeny suffered from low resolution (Schouls et al., 2003), and it was not a suitable approach to differentiate *S. intermedius* from *S. constellatus* (Issa et al., 2019). On the other hand, *cpn60* outperformed both 16S rRNA and *rpoB* in differentiation between individual strains (Olson et al., 2013). Results obtained from single locus genetic markers might not be as reliable as those obtained with multiple genetic loci. In fact, a multi-locus sequence analysis (MLSA) approach of concatenated sequences of seven housekeeping genes was shown to be more promising in identifying well-resolved species clusters (Bishop et al., 2009; Jensen et al., 2013). Bishop et al. (2009) collected seven housekeeping gene sequences from 420 streptococci to produce a viridans group database and developed a publicly available website and software for electronic taxonomy^1^. The seven targeted genes were *map* encoded for a methionine aminopeptidase, *pfl*, a pyruvate formate lyase, *ppaC*, an inorganic pyrophosphatase, *pyk*, a pyruvate kinase, *rpoB*, an RNA polymerase subunit, *soda*, a superoxide dismutase, and *tuf*, an elongation factor Tu (Bishop et al., 2009). Jensen et al. (2013) used the MLSA-based approach and performed a phylogenetic analysis of concatenated sequences of the seven housekeeping genes in 123 SAG isolates. As a result, they were able to differentiate between seven distinct and coherent clusters in SAG and showed that single gene analyses are often invalid as they suffered from allele sharing between species (Jensen et al., 2013).

The advent of WGS has provided, however, a better resolution and discriminatory power between closely related organisms at the species and strain levels by looking into the accumulation of single-nucleotide polymorphisms (SNPs), core genome conservation, virulence potential, horizontally transferred genetic material, and microevolution within the host (Olson et al., 2013). This approach was better at comparing and identifying the isolates at the species and strain level (Issa et al., 2019). Although 42 genome assemblies of *S. intermedius* are currently available on the NCBI database (last accessed on 08/05/2019), *S. intermedius* still remains under-represented compared to other SAG members and other medically relevant streptococci (Figure 1B).

## Clinical Cases

Case reports of *S. intermedius* infections were collected from PubMed (last accessed on 08/05/2019) occurring over a 23-year period (1996–2019). A total of 101 cases with available patient metadata were retained, 85.1% (*n* = 86) of which were reported after the year of 2015, 12.9% (*n* = 13) between 2001 and 2014, and 2.0% (*n* = 2) prior to 2001. The following demographic and clinical data was obtained from the literature: patient’s age, sex, year of infection (if not available the publication date was used), country of isolation, clinical profile (including previous relevant pathologies, surgeries, dental manipulation, etc.), symptoms, neurological results when available (including sensory losses, quadriplegia, numbness, weakness, movement restriction etc.), imaging results [mainly computed tomography (CT)], isolation source of the bacterium, coinfection with other bacteria, presence or absence of an abscess and its localization, prescribed antibiotics, and outcome of the disease (recovery or death) (Table 1 and Supplementary Table S1).

**TABLE 1:** Characteristics of included *S. intermedius* case reports.

**Parameter**	**Description**
Cases	101
Age	5–83 (Average: 44.8)
Sex	63.1% M; 36.9% F
Year	1996–2019
Country	24 countries
Clinical profile	Including dental manipulation, sinusitis, etc.
Symptoms	Including fever, headaches, etc.
Neurological results	Including sensory losses, numbness, etc
Imaging results	CT scan, MRI
Isolation source	Including blood, brain, etc.
Co-infection	Bacterial, polymicrobial
Abscess	98.1% P; 1.9% A
Abscess localization	Including brain, lung, liver, etc.
Treatment	Including abscess drainage, surgery, etc.
Prescribed antibiotics	Various, including combinations
Outcome	92.2% recovery; 7.8% death

**M, male; F, female; CT, computerized tomography; MRI, magnetic resonance imaging; P, present; A, absent.**

### Patient Age, Sex, and Location

**FIGURE 2:**
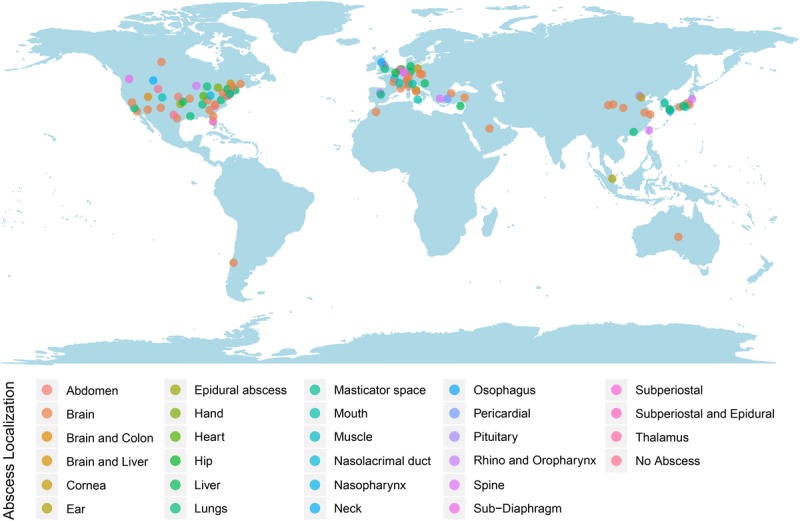
Worldwide reported *S. intermedius* cases. Dots were colored according to the abscess localizations.

**FIGURE 3:**
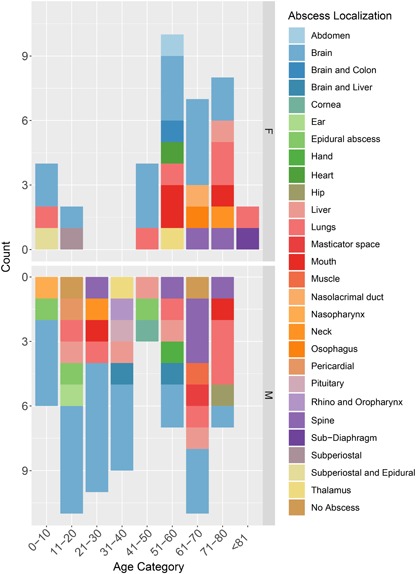
Distribution of case reports based on patients’ age categories, sex, and abscess localization. M, male; F, Female.

Patients’ ages ranged from 5 to 83 years old. The mean patient age was 45± 24 years old. Most cases of S. intermedius infection were identified in patients within older age groups of 61–70 (17.8%; *n* = 18), followed by 51–60 (16.8%; *n* = 17) and 14.9% (*n* = 15) for 71–80-year-old patients. Only two cases (2.0%) were >80 years old. The remaining of the cases were 0–10 years old (9.9%; *n* = 10), 11–20 years old (12.9%; *n* = 13), 21–30 years old (9.9%; *n* = 10), 31–40 years old (8.9%; *n* = 9), and 41–50 years old (6.9%; *n* = 7). Notably, the cases included approximately twice as many males as females (M: 63.4%; *n* = 64 and F: 36.6%; *n* = 37), with the mortality rate being 6.9% (*n* = 7). Most reported cases were from the United States (38.6%; *n* = 39), followed by Europe (32.7%; *n* =32), East Asia (19.8%; *n* = 20), the Middle East and North Africa (4.9%; *n* = 5), Canada (3.0%; *n* = 3), and Australia and Chile (1.0%; *n* = 1, each) (Figure 2). No age- or geo-specific pattern for abscess localization were detected (Figure 2, 3).

In accordance with our findings, a retrospective analysis of a hospital’s admissions between 2004 and 2009 in Australia showed male predominance of *S. intermedius* infections comprising 67% (*n* = 6) of all cases as opposed to 33% (*n* = 3) of infections affecting females. The mean patient age was reported to be higher (average = 59.8 years) (Junckerstorff et al., 2014).

### Symptoms

Upon admission, most patients showed primarily symptoms of intermittent fever and consistent headaches. These symptoms classically imply a potential brain abscess (Brouwer et al., 2014). Nonetheless, proper imaging techniques are needed to diagnose and localize a brain abscess. Blood tests for the C-reactive protein and WBC count were elevated and indicative of an infection.

### Risk Factors

Underlying risk factors for *S. intermedius* infections were detected among patients. Most patients experienced a previous dental manipulation (18.8%; *n* = 19) or suffered from sinusitis (11.9%; *n* = 12). Other risk factors included a history of diabetes (7.9%; *n* = 8), heavy alcohol consumption (7.9%; *n* = 8) congenital heart disease and heart-related conditions (7.9%; *n* = 8), and cancer (6.9%; *n* = 7). Patients also reported of undergoing surgery (4.9%; *n* = 5), having asthma (4.0%; *n* = 4), suffering from falls (4.0%; *n* = 4), smoking (4.0%; *n* = 4), and use of drugs (3.0%; *n* = 3).

A previous study identified the main risk factors of SAG infections (Suzuki et al., 2016). Solid tumors were the most comorbid (32.1%, *n* = 25), followed by diabetes mellitus (18.0%, *n* = 14), heart failure, liver disease, dementia (each 12.8%, *n* = 10), hemiplegia, cerebral vascular disease (each 11.5%, *n* = 9), myocardial infarction (7.7%, *n* = 6), collagen disease, peripheral artery disease (each 6.4%, *n* = 5), chronic respiratory disease (5.1%, *n* = 4), peptic ulcer disease, chronic kidney disease (3.9%, *n* = 3), and leukemia (1.3%, *n* = 1) (Suzuki et al., 2016). In light of these reports and our findings, more attention has to be given to dental manipulations, sinusitis, and diabetes mellitus as underlying factors for *S. intermedius* infections.

### Abscess Formation and Detection

*S. intermedius* was mainly isolated from pus (34.7%; *n* = 35), brain (16.8%; *n* = 17), blood (14.9%; *n* = 15), and cerebrospinal fluid (6.9%; *n* = 7). Isolation from the cornea, ear, mouth, nose, esophagus, heart, and lung were also reported. Almost all infections (98.0%, *n* = 99) were associated with abscess development, detected through various imaging approaches including computed tomography (CT), scans and magnetic resonance imaging (MRIs). Brain abscesses were the most abundant (41.6%), followed by lung (12.9%) as well as liver and spine (7.9% each) (Figure 3).

Similarly, Kobo et al. (2017) reported greater *S. intermedius* isolation from pyogenic infections (86.1%, *n* = 31) as compared to non-pyogenic infections (13.9%, *n* = 5). Another study correlated specific body sites with *S. intermedius* infection which prominently affected the central nervous system (62.1%, *n* = 18), followed by abdominal or pelvic sites (13.8%, *n* = 4), skin, soft tissues, bone (13.8%, *n* = 4), and head and neck infections (10.3%, *n* = 3) (Whiley et al., 1992).

### Treatment

Abscess drainage and surgery remained as the only interventions to limit the abscess and were performed in 32.7% (*n* = 33) and 22.8% (*n* = 23) of the cases, respectively. The use of antibiotics was mentioned in 78.2% (*n* = 79) of the studied case reports. Antibiotic treatments were administered for up to 6 weeks after discharge from the hospital. A variety of antibiotics were prescribed to treat the patients with combination therapies prescribed in 77.2% (*n* = 61), and a single antibiotic treatment was prescribed in 25.3% (*n* = 20) of the cases. Upon admission, most commonly prescribed antibiotic regimens were a combination of ceftriaxone and metronidazole alone (6.3%; *n* = 5) or the latter two combined with vancomycin (6.3%; *n* = 5). Interestingly, the prescribed antibiotic regimen was changed during the course of infection for 32.9% (*n* = 26) of the patients; the antibiotic treatment prescribed upon hospital admission was most frequently changed to include metronidazole (30.8%; *n* = 8), ceftriaxone (19.2%; *n* = 5), or meropenem (15.4%; *n* = 4). During the entire course of treatment, the most commonly prescribed antibiotic was metronidazole (44.3%; *n* = 35) followed by: ceftriaxone (39.2%; *n* = 31), vancomycin (22.8%; *n* = 18), penicillin (15.2%; *n* = 12), clindamycin (12.7%; *n* = 10), meropenem (13.9%; *n* = 11), and ampicillin (12.7%; *n* = 10). Subsequently patients were commonly exposed to 1–6 antibiotics during the duration of treatment with 4.9% (*n* = 4), 8.6% (*n* = 7), 11.1% (*n* = 9), 17.2% (*n* = 12), 28.4% (*n* = 23), and 25.9% (*n* = 21) patients being prescribed six, five, four, three, two, or one antibiotic, respectively. With no published guidelines or recommendations to treat SAG, the choice and duration of treatment remains unclear (De Louvois et al., 2000; Yakut et al., 2015). Nevertheless, the excessive use of antibiotics is alarming and favors the emergence of drug resistance especially with vancomycin and carbapenems (meropenem) (Mohammadi et al., 2019).

Prior retrospective studies by Clarridge et al. (2001) suggested the necessity of CT-guided aspiration or extensive surgery in both the diagnosis and treatment of *S. intermedius*. Furthermore, patients in this study were administered penicillin or its enhanced form as a single-drug therapy or a combination of drugs in cases of polymicrobial infections. Subsequent effective treatment outcomes suggested microbial susceptibility to the antibiotic used (Clarridge et al., 2001).

### Co-infection

Co-infection with microorganisms other than *S. intermedius* was detected in 29.7% of the studied cases (*n* = 30). Other SAG members were commonly detected such as *S. anginosus* (10.0%; *n* = 3) and *S. constellatus* (13.3%; *n* = 4) in addition to other streptococci, such as non-enterococcal group D streptococci, *S. viridans*, *S. salivarus*, and *S. pneumoniae*. Other commonly detected organisms included *Actinomyces* species (13.3%; *n* = 4) and *Fusobacterium* species (6.7%; *n* = 2). *Staphylococcus* species were found in 13.3% (*n* = 4) of co-infection cases.

Previously Furuichi and Horikoshi (2018), revealed a higher occurrence of polymicrobial infections (71.4%, *n* = 10) and included anaerobic microorganisms, mainly *Peptostreptococcus spp.* (14%, *n* = 2), *Bacteroides spp.* (7%, *n* = 1), *Prevotella spp.* (7%, *n* = 1), and *Porphyromonas spp.* (7%, *n* = 1). Other gram-positive bacteria were isolated and commonly identified as *Staphylococcus* species (21%, *n* = 3), cocci (29%, *n* = 4), and rods (7%, *n* = 1). Bacterial cultures also revealed gram-negative cocci (21%, *n* = 3), *Haemophilus influenzae* (14%, *n* = 2), and *Eikenella corrodens* (7%, *n* = 1).

## Virulence and Abscess Formation

**FIGURE 4:**
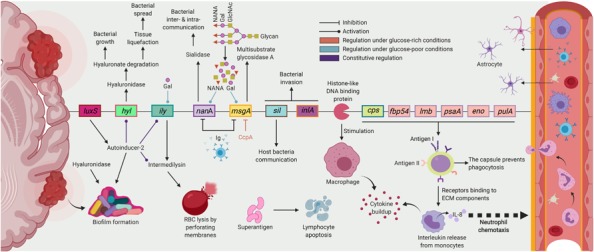
Major *S. intermedius* virulence factors and regulation of gene expression involved in brain abscess formation. Following *S. intermedius* infection, tissue damage is caused by the hydrolytic activity of hyaluronidase (*hyl*) and proinflammatory cytokine build-up from the binding of surface proteins to ECM components and macrophage stimulation by histone-like DNA binding proteins (HLPs). *Hyl* also degrades the hyaluronate constituent of connective tissues, which serves as a bacterial nutrient. Antigens I/II surface proteins (*fbp54* and *lmb*) bind to fibronectin and laminin of the ECM, which stimulates interleukin 8 release from monocytes followed by neutrophil chemotaxis. Adhesion is also mediated by surface proteins (*psaA*), enolase (*eno*), and pullulanase (*pulA*). *S. intermedius* escapes host immunological defenses through the expression of lymphocyte-apoptotic superantigens and biofilm formation monitored by the product of the *luxS* gene, antoinducer-2, and hyaluronidase activity. Invasion is ensured by a complete Streptococcus Invasion locus (*sil*) system and a homolog of internalin A. Replication and abscess formation are allowed through the polysaccharidic capsular inhibition of phagocytic polymorphonuclear neutrophils. Intermedilysin (ILY) and sialidase A (NanA) are *S. intermedius*-specific virulence factors. ILY binds to the human complement regulator CD59 (hCD59) and creates pores in membranes of red blood cells allowing for necrosis. NanA releases sialic acid moieties from sugar chains present in the environment and the surface of bacteria to direct inter-bacterial and host-bacterial interactions. Regulation of gene expression depends on carbohydrate supply. Under glucose-poor conditions (light blue), multi-substrate glycosidase A (MsgA) and NanA degrade glycans in the human serum to release N-acetylneuraminic acid and galactose. Galactose inhibits the lactose phosphotransferase system repressor (LacR), an ILY silencer, resulting in a constitutive ILY expression and a positive feedback loop for MsgA and NanA expression. Under glucose-rich conditions (red), the catabolite control protein (CcpA) represses ILY expression via either binding to the *ily* promoter on the catabolite repressible element (cre) or controlling the concentration of *ily* transcriptional regulators. ILY, MsgA, and NanA activities are also reduced by immunoglobulins in the human plasma. The *luxS* pathway (violet) constitutively increase ILY and *hyl* expression and the ILY hemolytic activity but not that of hyaluronidase (figure created with BioRender).

SAG members have been identified as the most common organisms isolated from brain abscess (Prasad et al., 2006; Sibley et al., 2012), liver abscess (Sibley et al., 2010), and empyema (Ahmed et al., 2006). *S. intermedius* has been mostly associated with central nervous system infections, particularly brain abscesses (Whiley et al., 1992). Currently, the exact genetic components leading to brain abscess development in some strains of *S. intermedius*, but not others, are not well understood. The known factors contributing to *S. intermedius* invasion and brain abscess formation are summarized in Figure 4.

### Antigens I/II

The onset of *S. intermedius* brain abscess formation first begins with tissue damage that precedes bacterial colonization. This occurs by binding to human fibronectin and laminin components of the extracellular matrix (ECM) through antigens I/II surface proteins, and they are coded by *fbp54* and *lmb*, respectively (Baddour, 1994; Petersen et al., 2001). The immunological response to binding is manifested by Interleukin 8 (IL-8), released from monocytes, which in turn activates neutrophil chemotaxis and contributes to proinflammatory cytokine build-up causing tissue damage (Kielian et al., 2001; Petersen et al., 2001). Cytokines are also released from human macrophages upon stimulation by histone-like DNA binding proteins (HLPs), and this alters the nucleoid architecture to control gene transcription (Liu et al., 2008). Adhesion to the ECM is also facilitated by the surface protein (*psaA*), enolase (*eno*) for plasminogen binding, and pullulanase (*pulA*) (Issa et al., 2019). The PsaA protein ortholog in *S. pneumoniae* has been shown to bind to the adhesion molecule E-cadherin (Anderton et al., 2007). Also, α-Enolase is the major plasmin- and plasminogen-binding protein of streptococci and was associated with binding to mucin in *S. mutans* (Ge et al., 2004). Finally, *pulA* was linked to the strepadhesin activity of *S. pyogenes* (Hytönen et al., 2003).

### Hyaluronidase

*S. intermedius* further expresses hydrolytic enzymes responsible for tissue liquefaction and pus formation, such as hyaluronidase (*hyl*), chondroitin sulfatase, and deoxyribonuclease. Hyaluronidase is also a growth factor that mobilizes low molecular weight nutrients in the host to be used by this bacterium (Ruoff and Ferraro, 1987; Willcox et al., 1995; Takao et al., 1997). Consequently, higher hyaluronidase concentrations are observed in deeper abscesses as its expression depends on substrate availability of hyaluronate (Homer et al., 1997). Hyaluronidase facilitates bacterial and toxin spread in tissues through increased connective tissue permeability and decreased ground substance viscosity of the ECM by degrading the hyaluronate constituent of theses tissues (Hynes and Walton, 2000).

### Autoinducer-2 and Superantigens

Biofilm formation is also dependent on hyaluronidase activity and on autoinducer-2 (AI-2), a furanosyl borate diester signal molecule product of the *luxS* gene. AI-2 controls quorum-sensing communication, and, by that, the tendency for biofilm formation. *S. intermedius* pathogenesis is induced by biofilm formation, which also decreases antibiotic susceptibility and protects from host immunological defenses (Vendeville et al., 2005; Pecharki et al., 2008b). *S. intermedius* expresses additional defensive genes to oppose the human immune system, such as superantigens that cause lymphocyte apoptosis or proteins to arrest lymphocyte and fibroblast proliferation (Arala-Chaves et al., 1979; Proft and Fraser, 1998).

### The Capsule

A higher tendency for abscess formation is observed in encapsulated *S. intermedius* isolates (Brook and Walker, 1985). In fact, the polysaccharidic capsule of *S. intermedius* prevents phagocytosis, as it inhibits polymorphonuclear neutrophils from exerting a phagocytic function to allow for replication after arrival to the infection site and adherence. This inhibition depends on the concentration of capsular polysaccharides (Wessels, 1997; Kanamori et al., 2004).

### *Streptococcus* Invasion Locus *(sil)*

Bacterial invasion is assisted by a complete *Streptococcus* Invasion locus (*sil*) system detected in *S. intermedius* (Issa et al., 2019). The *sil* genes improve virulence and delay wound healing in *S. pyogenes* infections (Salim et al., 2008). Their activity was first identified in GAS members where a transposon insertion in the *sil* system attenuated virulence in a murine model (Hidalgo-Grass et al., 2002). Moreover, the *sil* system has been implicated in the regulation of microbe–microbe interactions at mucosal surfaces through putative bacteriocins that gave *S. intermedius* a competitive advantage (Mendonca et al., 2017).

### Intermedilysin and Sialidase A

Two *S. intermedius*-specific virulence factors, namely the intermedilysin (ILY) and sialidase A (NanA), are not found in other members of the SAG group and are preserved under purifying selection (Takao et al., 2010; Issa et al., 2019). Cholesterol-dependent cytolysin ILY does not bind cholesterol directly, which makes *S. intermedius* a strictly human-specific pathogen (Giddings et al., 2004). Instead, ILY binds its cell-surface receptor hCD59, which initiates pore complex formation in host cell membranes especially red blood cells causing their programmed necrosis (Giddings et al., 2004) and prevents binding to the C8 and C9 complement components, thereby inducing host cell lysis via the formation of the membrane attack complex (MAC) (LaChapelle et al., 2009).

NanA, on the other hand, contributes to pathogenicity by controlling inter-bacterial communication and host-bacterial interactions through releasing sialic acid moieties from sugar chains present in the environment and the surface of bacteria (Long et al., 2004; Orihuela et al., 2004; Takao et al., 2010).

## Regulation of Gene Expression

ILY activity is reciprocally regulated by either activation of *ily* gene expression or immunological inhibition by blood modulators according to carbohydrate availability.

### Glucose-Poor Conditions

In the absence of glucose, the metabolic degradation of glycans in the human serum by multi-substrate glycosidase A (MsgA) and NanA releases *N*-acetylneuraminic acid and galactose. The latter is an inhibitory monosaccharide of the lactose phosphotransferase system repressor (LacR) (Tomoyasu et al., 2013; Imaki et al., 2014). Being a transcriptional regulator from the GntR family, LacR normally binds to the *ily* promoter and represses transcription (Rooijen et al., 1992; Tomoyasu et al., 2013). Therefore, inactivation of LacR under galactose-rich conditions results in constitutive ILY stimulation, as seen by the increased toxicity toward hepatoma cells. MsgA and NanA expression is also upregulated by a positive feedback loop, which induces *S. intermedius* virulence (Tomoyasu et al., 2017). Notably, genomic analysis of a deep-seated abscess correlated a non-functional and mutated LacR to ILY overproduction (Tomoyasu et al., 2013). The expression pattern of ILY is, however, negatively affected by elevated concentrations of other utilizable sugars, including glucose, fructose, and maltose.

### Glucose-Rich Conditions

Under abundant glucose supply, transcriptional repression of ILY is achieved by the catabolite control protein (CcpA), which either binds to the *ily* promoter on the catabolite repressible element (cre) or controls the concentration of transcriptional regulators for *ily*. The dual inhibition pathways suggested the presence of two alternating carbohydrate-dependent types of growth, rapid-slightly virulent and slow-highly virulent, depending on environmental carbohydrate abundance or limitation, respectively (Tomoyasu et al., 2010). Carbohydrate association with *ily* gene expression suggested the presence of mechanisms controlling sugar concentrations (Tomoyasu et al., 2017). Given the importance of ILY in the increased virulence of this pathogen, further work is warranted to characterize the mechanisms involved and their correlation to pathogenicity and/or commensalism.

Sugar availability is also important during the early stages of infection; the ability of *S. intermedius* to survive dental restoration and obtain nutrients for survival has been linked to degradation of galactose, *N*-acetyl-D-glucosamine, and *N*-acetylneuraminic acid on serum glycoproteins from the pulp of the infected dentin (Paddick et al., 2005).

### Oxygen Availability

Differential growth kinetics and metabolic activities of *S. intermedius* are influenced by oxygen availability, as observed by Fei et al. (2016). *S. intermedius* showed an increased growth rate in an anaerobic environment, which could be prompted by an increased expression of genes acting in nucleoside and nucleotide metabolism. Furthermore, an upregulation of glycolysis-associated genes resulted in an increased glucose metabolism. An enhanced expression of the glycogen biosynthesis operon (*glgABCD*) was also observed, suggesting the storage of glucose in the form of glycogen (Fei et al., 2016).

Moreover, under anaerobic conditions, *S. intermedius* increased arginine turnover to carbamoyl phosphate via the arginine deiminase pathway, leading to an increase in pyrimidine *de novo* synthesis (Fei et al., 2016). As such, arginine consumption by *S. intermedius* limited nitric oxide production by host cells, favoring, as a result, its survival in the host (Stadelmann et al., 2013; Cusumano et al., 2014). Although the expression of virulence-associated genes was not related to the presence or absence of oxygen, *nanA* and *pulA* genes were upregulated under anaerobic conditions (Fei et al., 2016).

On the other hand, under aerobic conditions, genes encoding DNA-repair pathways, iron metabolism, and iron-sulfur clusters were upregulated (Fei et al., 2016).

Notably, bacterial membrane composition also varied to accommodate the differences in oxygen availability (Fei et al., 2016). These observed changes in the membrane merit further investigation to map their role in S. intermedius pathogenesis, if any.

### Immunological Inhibition

Immunoglobulins in the human plasma also have a regulative property in preventing ILY, MsgA, and NanA activities (Tomoyasu et al., 2017). ILY-silencing experiments via an anti-ILY antibody showed decreased *S. intermedius* cytotoxicity toward cancerous human liver cells (Sukeno et al., 2005). Efficiency of the immunoreaction relies on antibody concentration especially since partial neutralization of ILY activity in immunocompromised patients would exert a positive feedback mechanism on *ily* production further inducing infection rather than terminating it (Tomoyasu et al., 2017). Antibody concentration represents a form of adaptive immune mechanism since higher anti-ILY levels are observed in patients with past *S. intermedius* infections. Human plasma immunoglobulins that neutralize ILY could also neutralize MsgA and NanA activities, though to varying extents (Tomoyasu et al., 2017).

### The *luxS* Pathway

An additional *ily* and *hyl* level of regulation involves *luxS* (autoinducer-; AI-2). Mutations introduced in *luxS* led to a decrease in *ily* and *hyl* expression and attenuated ILY hemolytic activity, though not that of hyaluronidase. The disposition of membrane antigens I/II didn’t change as *luxS* does not control the expression of the relevant genes (*fbp54* for antigen I and *lmb* for antigen II) (Pecharki et al., 2008a). AI-2 mediated regulation also involves other factors, such as iron acquisition, carbohydrate metabolism, proteolytic and hemolytic activities, antibiotic production, and biofilm formation (Vendeville et al., 2005). The link between the *luxS* and regulation of carbohydrate metabolism is not yet clear. The role of *luxS* as a ‘chief gene’ might suggest its involvement in the control of sugar-sensing monitors at the base of carbohydrate-dependent regulation.

## Genome Insights

The *S. intermedius* genome is comprised of a single circular chromosome having an average size of 1.99 Mbp and includes roughly ∼1,700 coding genes and a 37.6% G + C content. Similar to other streptococci (Ajdiæ et al., 2002; Holden et al., 2009), no plasmid DNA have been reported in *S. intermedius* (Olson et al., 2013). At least two *S. constellatus* strains have been previously misidentified and deposited on NCBI as *S. intermedius*, F0395 (Jensen et al., 2013), and 567_SINT (Issa et al., 2019). The genome of *S. intermedius* consists of 1,327 core genes (≥ 99% of isolates), 991 shell genes present in two or more isolates (15% ≤ isolates < 95%), and 1,672 unique cloud genes specific to single isolates (0% ≤ isolates < 15%) (Issa et al., 2019). This is less than the 1,617 core genes found in *S. constellatus* (Olson et al., 2013).

Few studies have been performed on the whole-genome molecular characterization and comparative genome analysis of *S. intermedius* (Olson et al., 2013; Planet et al., 2013; Hasegawa et al., 2017; Issa et al., 2019). A genome announcement for the BA1 strain isolated from an intracranial abscess in a child was published by Planet et al. (2013). Olson et al. (2013) used an intra and inter species comparative genome analysis approach of seven SAG genomes and 59 publicly available *Streptococcus* genomes to clarify the phylogenetic relationships of these bacteria and supports their distinct species classification. Shared orthologous proteins suggested that the SAG genomes were most closely related to *S. gordonii* and *S. sanguinis* yet harbored a similar number of proteins within each COG category to other *Streptococcus* species. *S. intermedius* was mostly similar to *S. constellatus*. Mobile elements, primarily integrative conjugative elements, and bacteriophage were found to account for more than 10% of the SAG genomes with *S. anginosus* being the most variable species. *S. intermedius* and *S. constellatus*, on the other hand, shared greater than half of their virulence traits and with numerous potential virulence determinants in SAG.

Hasegawa et al. (2017) performed a comprehensive molecular characterization of *S. intermedius* pathogenicity based on the complete genome sequence and a murine subcutaneous abscess model with transcriptome and random transposon mutagenesis of TYG1620 strain isolated from a brain abscess in an infant. They reported the presence of a notable type VII secretion system (T7SS), two long repeat regions, and 19 ORFs for cell wall-anchored proteins (CWAPs). Transcriptome analysis in a murine subcutaneous abscess model suggested that the levels of expression of small hypothetical proteins similar to phenol-soluble modulin _1 (PSM_1), a staphylococcal virulence factor, significantly increased in the abscess model. Finally, Issa et al. (2019) performed a whole-genome comparative analysis of LAU_SINT associated with a brain abscess with 16 *S. intermedius* genomes in addition to *S. constellatus* and *S. anginosus*. No geographical correlation between pan-genome and SNPs-based analysis could be established. Four different T7SS modules (I–IV) located on various genomic island were described, and nisin resistance determinants were found in 47% of the isolates.

## Conclusion

*S. intermedius* is one of the most common pathogens associated with brain as well as liver abscesses and thoracic empyema. A search on PubMed Central revealed that SAG members are clearly underrepresented compared to other medically relevant streptococci. Patients with invasive *S. intermedius* infections have a significantly longer hospital stay and higher mortality rates than other SAG members. Dental manipulation or sinusitis are the two most important underlying risk factors for *S. intermedius* infections. Almost all infections are associated with abscess development, detected through various imaging approaches including CT, scans, and MRIs. *S. intermedius* brain abscess formation begins with tissue damage mediated by the binding to human fibronectin and laminin and the subsequent Il-8 release from monocytes, which activates neutrophil chemotaxis and contributes to the proinflammatory cytokine build-up causing tissue damage. *S. intermedius* expresses hydrolytic enzymes responsible for the tissue liquefaction and pus formation with higher hyaluronidase concentrations being observed in deeper abscesses. Biofilm formation is also dependent on hyaluronidase activity and on AI-2. ILY and NanA, two important virulence determinants, are not found in other members of the SAG group. ILY activity is regulated by *ily* gene expression and/or immunological inhibition by blood modulators depending on the availability of carbohydrates. Abscess drainage and surgery remained the only interventions to limit the abscess. A variety of antibiotics were prescribed to treat the patients with combination therapies being the dominant used approach. Upon admission, the most commonly prescribed antibiotic regimens were a combination of ceftriaxone and metronidazole alone or combined with vancomycin. The prescribed antibiotic regimen was changed most frequently to include metronidazole, ceftriaxone, or meropenem.

Carbohydrate association with *ily* gene expression and the correlation between a deep-seated abscess with a non-functional and mutated LacR warrant further studies to better characterize the mechanisms involved. Medical diagnostic laboratories could adopt *ily* PCR for a rapid and accurate identification and diagnosis in cases where patients present any of the previously stated risk factors and symptoms. In this context, improved guidelines for antibiotics drug administration can aid in preventing the random and excessive antibiotics usage in *S. intermedius* infections. Additional research is also required to elucidate mechanisms used by *S. intermedius* to cross the blood brain barrier and to design and implement targeted therapeutic approaches. Finally, the dependency of *S. intermedius* virulence on sugar availability requires further experimental testing, as it represents an alternative nutritional therapy based on dietary restriction of sugar intake.

## Summary

This review aimed at summarizing our current knowledge of *S. intermedius*, its various identification methods, known virulence factors, and regulation of gene expression involved in abscess development. Members of the SAG are part of the normal microbiota being found at various mucosal sites and are frequently encountered in invasive suppurative infections. SAG members share few common characteristics and have a variable Lancefield serogrouping. Different identification approaches were used with the most promising being the MLSA and WGS, with the WGS approach providing a better resolution and discriminatory power. Currently, 42 genome assemblies of *S. intermedius* are available on NCBI database, and *S. intermedius* still remains underrepresented compared to other SAG members and other medically relevant streptococci. A total of 101 cases with available patient’s metadata were retained in this review, which revealed a male predominance of *S. intermedius* infections with most patients showing upon admission intermittent fever and consistent headaches. Blood tests for the C-reactive protein and WBC count were elevated and indicative of an infection. The most important underlying risk factor and comorbidity was dental manipulation and sinusitis, respectively. Abscess drainage and surgery remained as the only interventions to limit the abscess, and antibiotic treatment prescribed upon hospital admission was most frequently changed to include metronidazole, which was the most commonly prescribed antibiotic. The onset of *S. intermedius* brain abscess formation first begins with tissue damage that precedes bacterial colonization, tissue liquefaction, and pus formation through hyaluronidase activity. Several virulence factors were detected, and ILY and NanA were specific to *S. intermedius*. Carbohydrate association with *ily* gene expression and the correlation between deep-seated abscess with a non-functional and mutated LacR warrant further studies to better characterize the mechanisms involved. Moreover, *ily* based PCR identification and improved guidelines for drug administration could aid in preventing the random and excessive antibiotics usage in treating *S. intermedius* infections. Additional research is also required to elucidate the mechanisms used by *S. intermedius* to cross the blood brain barrier and to determine the correlation between virulence and sugar availability.

## Author Contributions

ST performed the study conceptualization and performed the supervision. EI and TS performed the literature search, performed the writing, and the original draft preparation. ST, EI, and TS carried out the critical discussion, carried out the writing, reviewing, and editing. All authors have read and agreed to the published version of the manuscript.

## Conflict of Interest

The authors declare that the research was conducted in the absence of any commercial or financial relationships that could be construed as a potential conflict of interest.

## Footnotes

1 http://www.eMLSA.net
