# Supplementary material for: From Normal Flora to Brain Abscesses: A Review of Streptococcus intermedius
Source: Front Microbiol. 2020 May 7;11:826. doi: 10.3389/fmicb.2020.00826 (PMC7221147; doi:10.3389/fmicb.2020.00826)
Supplement: Supplementary file 1 [file Data_Sheet_1.docx]

**Data sheet 1. List of the references used for the analysis of *S. intermedius* case reports.** A summary of the analysed *S. intermedius* case reports is presented in Table S1.

Al Moussawi, H., Krzyzak, M., Awada, Z., and Chalhoub, J. M. (2018). *Streptococcus intermedius* Brain and Diverticular Abscesses After Dental Manipulation: A Case Report. *Cureus* 10.

Alam, A., Aragaki-Nakahodo, A., and Balestra, R. R. (2016). “Aspiration Meningitis: Mediastinitis And Brain Abscess Due To *Streptococcus intermedius*,” in *D49. LUNG INFECTION CASE REPORTS II: INFECTIONS OTHER THAN FUNGAL INFECTIONS* (American Thoracic Society), A7179–A7179.

Alzahrani, A. J., Habib, H. A., Elwadtidy, S. M. F., Mona, B. A., Somily, A. M., and others (2019). Brain abscess due to *Streptococcus intermedius* in a young patient secondary to partially treated sinusitis. *Journal of Nature and Science of Medicine* 2, 54.

Balanger, M., Hinet, M., Vacher, C., Bellaiche, N., Charrier, J.-L., and Millot, S. (2017). Osteomyelitis of the mandible after dental implants in an immunocompetent patient. *Case reports in dentistry* 2017.

Benadjaoud, Y., Klopp-Dutote, N., Choquet, M., Brunel, E., Guiheneuf, R., and Page, C. (2017). A case of acute clival osteomyelitis in a 7-year-old boy secondary to infection of a Thornwaldt cyst. *International journal of pediatric otorhinolaryngology* 95, 87–90.

Benou, C., Walter, B. M., Schlitter, M. A., Wilhelm, D., Neu, B., and Schmid, R. M. (2016). Gastrointestinal stromal tumor as entry port for S. intermedius causing bacteremia and multiple liver abscesses. Case report and review of literature. *Zeitschrift für Gastroenterologie* 54, 245–249.

Butskiy, O., Remillard, A., and Kozak, F. K. (2017). Forehead swelling in a 10-year-old male: A case report. *Current therapy for primary varicose veins*, 407.

Catalya, S., Komal, B., Tulpule, S., Raoof, N., and Sen, S. (2017). Isolated *Streptococcus intermedius* pulmonary nodules. *IDCases* 8, 48–49.

Cavrić, G., Nasabain, K., Jurić, K., Prkačin, I., BARTOLEK HAMP, D., and DVORŠČAK, O. (2015). Liver abscess caused by Actinomyces odontolyticus, Abiotrophia species, Haemophilus parainfluenzae, Streptococcus anginosus i *Streptococcus intermedius*-case report. *Signa vitae: journal for intesive care and emergency medicine* 10, 72–73.

Chengsupanimit, T., Sundaram, B., and Kane, G. C. (2019). Septic Pulmonary Embolism: A Case Series, Proposed Set of Diagnostic Criteria, and Review of the Literature. *Clinical Pulmonary Medicine* 26, 24–26.

Cheta, J. (2016). An atypical presentation of Lemierre’s syndrome with eye and facial pain: A case report with *Streptococcus intermedius*. *International Journal of Case Reports and Images (IJCRI)* 7, 848–852.

Cho, M., Snyder, C., Rivard, D., and Lim, J. (2017). Multiple giant mid-thoracic esophageal diverticula in a teenager. *Journal of Pediatric Surgery Case Reports* 27, 28–31.

Cillo Jr, J. E., and Barbosa, N. (2019). Adalimumab-related dental implant infection. *Journal of Oral and Maxillofacial Surgery* 77, 1165–1169.

Crespo, G.M., Martinez, A.F., and Gonzales, C.F.J. (2016). Destroyed lung complicated with empyema. *Imaging in Medicine* 8, 113-115.

Dandurand, C., Letourneau, L., Chaalala, C., Magro, E., and Bojanowski, M. W. (2016). Pyogenic ventriculitis as clinical presentation of diverticulitis. *Canadian Journal of Neurological Sciences* 43, 576–577.

D’Antico, C., Hofer, A., Fassl, J., Tobler, D., Zumofen, D., and Goettel, N. (2016). Case Report: Emergency awake craniotomy for cerebral abscess in a patient with unrepaired cyanotic congenital heart disease [version 1; referees: awaiting peer review].

Denby, K. J., Byrne, R. D., and Gómez-Duarte, O. G. (2017). *Streptococcus intermedius*: an unusual case of purulent pericarditis. *Case reports in infectious diseases* 2017.

Doan, N., Nguyen, H., Luyuan, L., Shabani, S., Gelsomino, M., and Johnson, V. (2018). Good outcomes with the intraventricular vancomycin therapy in a patient with ruptured brain abscesses. *Asian journal of neurosurgery* 13, 396.

El Kamouni, Y., Arsalane, L., Allali, A., Beddou, G., and Zouhair, S. (2017). Rare case of pyogenic brain abscess in a immunocompetent children caused by *Streptococcus intermedius*. *children* 5, 7.

Fadda, G., Berrone, M., Crosetti, E., and Succo, G. (2016). Monolateral sinonasal complications of dental disease or treatment: when does endoscopic endonasal surgery require an intraoral approach? *Acta Otorhinolaryngologica Italica* 36, 300.

Fisher, C., Puello, F., Ferm, S., Rubin, M., and Schnall, H. A. (2017). A Case of Sub-Diaphragmatic Abscess after Injection of Botulinum Toxin to Treat Achalasia. *ACG Case Rep J* 4. doi:[10.14309/crj.2017.119](https://doi.org/10.14309/crj.2017.119).

Gaffar, S., Birknes, J. K., and Cunnion, K. M. (2018). Trichophyton as a Rare Cause of Postoperative Wound Infection Resistant to Standard Empiric Antimicrobial Therapy. *Case reports in pediatrics* 2018.

Gan, J. Y., Tan, H. K. K., and Koh, L. H. (2017). Luc’s abscess masquerading as severe otitis externa. *International Journal of Pediatric Otorhinolaryngology Extra* 16, 10–13.

Gjeorgjievski, M., Reddy, N., Stecevic, V., and Cappell, M. S. (2018). Abdominal Abscess Related to Endoscopically Placed AspireAssist® Device. *ACG case reports journal* 5.

Glen, P., and Morrison, J. (2016). Diffuse descending necrotising mediastinitis and pleural empyema secondary to acute odontogenic infection resulting in severe dysphagia. *Case Reports* 2016.

Green, A., Flower, E., and New, N. (2001). Mortality associated with odontogenic infection! *British dental journal* 190, 529–530.

Greeneway, G. P., West, J. L., Couture, D. E., and Hsu, W. (2018). Cerebral Abscess in Young Adult with Hypoplastic Left Heart Syndrome. *World neurosurgery* 116, 201–204.

Guignard, N., Roujeau, T., Saumet, L., Gascou, G., Mondain, M., and Akkari, M. (2018). Sphenoidal sinogenic extradural empyema associated with juvenile myelomonocytic leukemia. *International journal of pediatric otorhinolaryngology* 115, 45–48.

Guillamet, L. J. V., Malinis, M. F., and Meyer, J. P. (2017). Emerging role of Actinomyces meyeri in brain abscesses: A case report and literature review. *IDCases* 10, 26–29.

Gupta, S., and Merchant, S. S. (2012). Lemierre’s Syndrome: Rare, but Life Threatening—A Case Report with *Streptococcus intermedius*. *Case Rep Med* 2012. doi:[10.1155/2012/624065](https://doi.org/10.1155/2012/624065).

Hameed, S., Singh, J., Tricia, L. B., Machado, A., Ruggieri, P., and Mehta, A. C. (2017). Conglomerate mediastinal mass of a different etiology. *Oxford medical case reports* 2017, omx072.

Hannoodi, F., Ali, I., Sabbagh, H., and Kumar, S. (2016). *Streptococcus intermedius* causing necrotizing pneumonia in an immune competent female: a case report and literature review. *Case reports in pulmonology* 2016.

Heckmann, J. G., Ernst, S., Scher, B., and Meyer, B. (2018). Rapidly growing thalamic abscess. *The Neurohospitalist* 8, 44.

Heckmann, J. G., and Pauli, S. U. (2015). Epidural abscess after dental extraction. *Age and ageing* 44, 901–901.

Höhne, J., Brawanski, A., and Schebesch, K.-M. (2017). Fluorescence-guided surgery of brain abscesses. *Clinical neurology and neurosurgery* 155, 36–39.

Honnorat, E., Seng, P., Riberi, A., Habib, G., and Stein, A. (2016). Late infectious endocarditis of surgical patch closure of atrial septal defects diagnosed by 18F-fluorodeoxyglucose gated cardiac computed tomography (18F-FDG-PET/CT): a case report. *BMC research notes* 9, 416.

Hu, H., Guo, L., Wu, H., Feng, W., Chen, T., and Liu, G. (2019). Evaluation of next-generation sequencing for the pathogenic diagnosis of children brain abscesses. *Journal of Infection* 78, 323–337.

Ioannou, A., Xenophontos, E., Karatsi, A., Petrides, C., Kleridou, M., and Zintilis, C. (2016). Insidious manifestation of pyogenic liver abscess caused by *Streptococcus intermedius* and Micrococcus luteus: a case report. *Oxford medical case reports* 2016, 1–3.

Ishimoto, H., Yatera, K., Uchimura, K., Oda, K., Takenaka, M., Kawanami, T., et al. (2015). A serious mediastinum abscess induced by endobronchial ultrasound-guided transbronchial needle aspiration (EBUS-TBNA): a case report and review of the literature. *Internal Medicine* 54, 2647–2650.

Juárez Escalona, I., Díaz Carandell, A., Aboul-Hons Centenero, S., Monner Diéguez, A., Marí Roig, A., Arranz Obispo, C., et al. (2007). Lemierre syndrome associated with dental infections: report of one case and review of the literature. *Medicina Oral, Patología Oral y Cirugía Bucal (Internet)* 12, 394–396.

Kaga, A., Higo, R., Yoshikawa, H., Yokoi, N., Haruyama, T., Komatsu, H., et al. (2017). A case of multiple empyema caused by *Streptococcus intermedius*. *Auris Nasus Larynx* 44, 745–748.

Kamar, F. and Dhingra, V. (2015). A *Streptococcus intermedius* Brain Abscess Causing Obstructive Hydrocephalus and Meningoventriculitis in an Adult Patient With Chronic Granulomatous Disease. *Canadian Journal of General Internal Medicine* 10.

Kaye, I. D., and Protopsaltis, T. S. (2016). Cervical facet joint infection and associated epidural abscess with *Streptococcus intermedius* from a dental infection origin: a case report and review. *Bulletin of the NYU Hospital for Joint Diseases* 74, 237–237.

Khaja, M., Adler, D., and Lominadze, G. (2017). Expressive aphasia caused by *Streptococcus intermedius* brain abscess in an immunocompetent patient. *International medical case reports journal* 10, 25.

Kikkawa, D. O., Heinz, G. W., Martin, R. T., Nunery, W. N., and Eiseman, A. S. (2002). Orbital cellulitis and abscess secondary to dacryocystitis. *Archives of Ophthalmology* 120, 1096–1099.

Ko, I.-C., Yoon, K.-H., Park, K.-S., Cheong, J.-K., Bae, J.-H., Lee, K.-W., et al. (2015). An unusual abscess formation in the masticator space after acupressure massage: a case report. *Journal of the Korean Association of Oral and Maxillofacial Surgeons* 41, 52–56. doi:[10.5125/jkaoms.2015.41.1.52](https://doi.org/10.5125/jkaoms.2015.41.1.52).

Korovessis, P., Repantis, T., Vitsas, V., and Vardakastanis, K. (2013). Cervical spondylodiscitis associated with oesophageal perforation: a rare complication after anterior cervical fusion. *European Journal of Orthopaedic Surgery & Traumatology* 23, 159–163.

Kuzaytepe, E. Ç., Karaaslan, A., Akın, Y., Hiçdönmez, T., Çiftçi, Ö., Meriç, İ., et al. (2016). Brain Abscess due to *Streptococcus intermedius* Secondary to Tetralogy of Fallot in a Child: A Case Report. *South Clin Ist Euras* 27, 246–249.

Lukassen, J. N. M., Aalbers, M. W., Coppes, M. H., and Groen, R. J. M. (2019). Cervical spondylodiscitis following cricopharyngeal botulinium toxin injection. *European annals of otorhinolaryngology, head and neck diseases* 136, 313–316.

Mashiko, R., Taguchi, S., Tobita, T., and Shibata, Y. (2017). Intracranial infection caused by minor skin contusion associated with previous craniotomy. *BMJ Case Rep* 2017. doi:[10.1136/bcr-2016-217833](https://doi.org/10.1136/bcr-2016-217833).

Matamala, J. M., Núñez, C., Ogrodnik, R., and Cartier, L. (2013). Cerebritis bifrontal y absceso cerebral por Streptococcus del grupo anginosus, en paciente previamente sano: Caso clínico. *Revista médica de Chile* 141, 109–113.

Michailidou, D. (2016). Acute necrotizing herpetic pleuritis: case report. *Reactions* 1607, 348–25.

Mortagy, M., Lutwick, L., and Breisach, S. (2017). Cryptococcal Empyema: A Rare Manifestation of Disease. *Research Day*. Available at: <https://scholarworks.wmich.edu/medicine_research_day/105>.

Nagai, M. Y., Miskimen, R., and Matthew Rossen DMD, M. D. (2018). Sequential Intracranial and Pulmonary Abscesses with S. intermedius as an Extension of Severe Maxillofacial Infection: A Case Report and Review of the Literature. *New York State Dental Journal* 84, 30–33.

Nagoshi, N., Shioda, M., Yorimitsu, E., and Yagi, M. (2018). Delayed adjacent level spondylodiscitis after initial surgery with instrumented spinal fusion: a report of three cases and review of the literature. *Case reports in orthopedics* 2018.

Nayfe, R., Ascha, M. S., and Rehmus, E. H. (2017). Esophageal Squamous Cell Carcinoma Presenting with *Streptococcus intermedius* Cerebral Abscess. *Case reports in pathology* 2017.

Neumayr, A., Kubitz, R., Bode, J., Bilk, P., and Häussinger, D. (2010). Multiple liver abscesses with isolation of *Streptococcus intermedius* related to a pyogenic dental infection in an immuno-competent patient. *European journal of medical research* 15, 319.

Noguchi, S., Yatera, K., Kawanami, T., Yamasaki, K., Fukuda, K., Naito, K., et al. (2014). Pneumonia and empyema caused by *Streptococcus intermedius* that shows the diagnostic importance of evaluating the microbiota in the lower respiratory tract. *Internal Medicine* 53, 47–50.

Öcal Demir, S., Kepenekli Kadayifci, E., Akkoç, G., Yakut, N., ŞAHİN, Y., DAĞÇINAR, A., et al. (2017). Brain Abscess Due to Aggregatibacter aphrophilus and *Streptococcus intermedius*: A Case Report. *Journal of the Child/Cocuk Dergisi* 17.

Pasquini, L., Espagnet, M. C. R., Esposito, G., De Vito, R., Messina, R., Gaspari, S., et al. (2018). Langerhans’ Cell Histiocytosis Mimicking a Pott Puffy Tumor. *Journal of pediatric hematology/oncology* 40, e182–e184.

Paul, A. B. M., Lary Simms, A. E. P., Yorke, J., and Schmidseder, C. (2016). A fatal case of empyema due to *Streptococcus intermedius* associated pneumonia masquerading as acute pancreatitis in an otherwise healthy middle-aged woman. *Int J Case Rep Images* 7, 524–528.

Pompucci, A., Bonis, P. D., Sabatino, G., Federico, G., Moschini, M., Anile, C., et al. (2007). Cranio-Spinal Subdural Empyema due to S. Intermedius: a Case Report. *Journal of Neuroimaging* 17, 358–360. doi:[10.1111/j.1552-6569.2007.00084.x](https://doi.org/10.1111/j.1552-6569.2007.00084.x).

Prášil, P., Boštíková, V., Hermannova, Z., and Plíšek, S. (2018). Invasive primary intracerebral infections in women caused by *Streptococcus intermedius* manifesting as purulent meningitis and intracerebral abscess. *Česká a slovenská neurologie a neurochirurgie* 81.

Quast, M. B., Carr, C. M., and Hooten, W. M. (2017). Multilevel lumbar spine infection due to poor dentition in an immunocompetent adult: a case report. *Journal of medical case reports* 11, 328.

Rainwater, E., Gupta, V., Vivekanandan, R., and Gorby, G. (2018). Maxillary Teeth Abscesses Result in Atypical Liver Abscesses. *Cureus* 10.

Ramhmdani, S., and Bydon, A. (2017). *Streptococcus intermedius*: an unusual cause of spinal epidural abscess. *Journal of Spine Surgery* 3, 243.

Reddy, S., Singh, K., and Hughes, S. (2018). Liver Abscesses Caused by *Streptococcus intermedius* in an Immunocompromised Patient. *Cureus* 10.

Ren, Y., Deschler, D. G., Sajed, D., and Durand, M. L. (2018). Case of late‐onset, relapsing surgical site infection related to a venous coupler placed during free flap reconstruction for major head and neck cancer. *Head & neck* 40, E29–E32.

Richardson, C., and Chhabra, N. (2018). Intranasal migration of a 35-year-old orbital plate presenting as unilateral epiphora. *Otolaryngology Case Reports* 9, 37–38.

Schuring, C., Trump, M., Sodhi, A., and Kadaria, D. (2017). “Emergent Purulent Pericarditis Presenting As Diabetic Ketoacidosis,” in *A56. CRITICAL CARE CASE REPORTS: CARDIOVASCULAR DISEASE I* (American Thoracic Society), A1928–A1928.

Sharma, P., Sharma, S., Gupta, N., Kochar, P., and Kumar, Y. (2017). Pott puffy tumor. in *Baylor University Medical Center Proceedings* (Taylor & Francis), 179–181.

Shibuya, H., Ikehara, H., Andoh, K., Horii, T., Moriyama, M., Yamao, K., et al. (2019). Endoscopic Ultrasound-guided Drainage of a Mediastinal Abscess Caused by an Ingested Fish Bone. *Internal Medicine* advpub. doi:[10.2169/internalmedicine.1992-18](https://doi.org/10.2169/internalmedicine.1992-18).

Shiu, S.-I., Lee, B.-J., Chen, H.-C., Lin, Y.-H., and Wang, C.-Y. (2014). Holospinal epidural abscess complicated with bilateral psoas muscle abscess. *The Spine Journal* 14, 1072–1073.

Simonin, A., Passaplan, C., Rusconi, A., Colin, V., Erard, V., Stauffer, E., et al. (2018). Pott’s puffy tumor presenting as a frontal swelling under a Swiss army helmet. *Clinical neurology and neurosurgery* 173, 115–117.

Sun, S., Yuan, G., Zhao, G., Chen, H., and Yu, B. (2010). Endophthalmitis caused by Phialophora verrucosa and *Streptococcus intermedius*: a case report. *Medical mycology* 48, 1108–1111.

Tanaka, Y., Takaya, K., Yamamoto, G., Shinzato, I., and Takafuta, T. (2015). Solitary Pyomyositis of the Left Rhomboideus Muscle Caused by Streptococcus anginosus and *Streptococcus intermedius* in an Immunocompetent Person. *Case reports in infectious diseases* 2015.

Tigen, E. T., Sari, I., Ak, K., Sert, S., Tigen, K., and Korten, V. (2015). Giant Purulent Pericarditis with Cardiac Tamponade Due to S treptococcus intermedius Rapidly Progressing to Constriction. *Echocardiography* 32, 1318–1321.

Tingate, C., and Alexander, H. (2019). Primary intraventricular abscess of the third ventricle. *Journal of Clinical Neuroscience* 65, 154–157.

Trück, J., Thompson, A., Dwivedi, R., Segal, S., Anand, G., and Kelly, D. F. (2015). Nonotogenic skull Base osteomyelitis in children: two Cases and a Review of the literature. *The Pediatric infectious disease journal* 34, 1025–1027.

Tsuang, F.-Y., Lin, Y.-T., Yen, D. H.-T., Teng, L.-J., and Tsai, J.-C. (2016). Rapid identification of *Streptococcus intermedius* by multiplex PCR one week before.

Uy, N., Thiagarajan, P., and Musher, D. M. (2015). Cephalosporin side chain idiosyncrasies: a case report of ceftriaxone-induced agranulocytosis and review of literature. in *Open forum infectious diseases* (Oxford University Press).

Van der Poel, N., Hansen, F., Georgalas, C., and Fokkens, W. (2016). Minimally invasive treatment of patients with Pott’s puffy tumour with or without endocranial extension-a case series of six patients: Our Experience. *Clin Otolaryngol* 41, 596–601.

Viviano, M., and Cocca, S. (2018). Multiple brain abscesses after professional tooth cleaning: Case report and literature review. *Journal of stomatology, oral and maxillofacial surgery* 119, 432–435.

Wagner, K. W., Schön, R., Schumacher, M., Schmelzeisen, R., and Schulze, D. (2006). Case report: brain and liver abscesses caused by oral infection with *Streptococcus intermedius*. *Oral Surgery, Oral Medicine, Oral Pathology, Oral Radiology, and Endodontology* 102, e21–e23.

Wallace, S., Deisher, D., and Harrison, T. (2017). Diverticulitis Resulting In Brain Abscess: A Case Report. *World Journal of Colorectal Surgery* 6. Available at: <https://scholarlyworks.lvhn.org/surgery/1370>.

Wang, Z., Gao, L., Zhou, X., Guo, X., Wang, Q., Lian, W., et al. (2018). Magnetic resonance imaging characteristics of pituitary abscess: a review of 51 cases. *World neurosurgery* 114, e900–e912.

Wei, L., Wang, F., and Chen, S. (2015). A late diagnosed case of Spontaneous esophageal perforation in an elderly patient. *International journal of clinical and experimental medicine* 8, 11594.

Wen, A. (2018). Cerebral Abscess. *Anesth Med Part J: AMPJ-122. DOI* 10.

Yamamoto, M., Fukushima, T., Ohshiro, S., Go, Y., Tsugu, H., Kono, K., et al. (1999). Brain abscess caused by *Streptococcus intermedius*: two case reports. *Surgical neurology* 51, 219–222.

Yang, C.-S., Zhang, L.-J., Sun, Z.-H., Yang, L., and Shi, F.-D. (2018). Acute prevertebral abscess secondary to intradiscal oxygen–ozone chemonucleolysis for treatment of a cervical disc herniation. *J Int Med Res* 46, 2461–2465. doi:[10.1177/0300060518764186](https://doi.org/10.1177/0300060518764186).

Zafar, S. Z., Pervin, N., Manthri, S., and Bhattarai, M. (2016). Multiple Brain Abscesses in an immunocompetent patient with factor V Leiden mutation. *Journal of investigative medicine high impact case reports* 4, 2324709616683724.

Zeller, V., Dedome, D., Lhotellier, L., Graff, W., Desplaces, N., and Marmor, S. (2016). Concomitant multiple joint arthroplasty infections: report on 16 cases. *The Journal of arthroplasty* 31, 2564–2568.

Zhabokritsky, A., Lam, P. W., and Salit, I. E. (2017). *Streptococcus intermedius* osteomyelitis of the radius. *Official Journal of the Association of Medical Microbiology and Infectious Disease Canada* 2, 38–40.
